# Transformational leadership and employee performance: the mediating role of affective organizational commitment

**DOI:** 10.3389/fpsyg.2026.1891273

**Published:** 2026-09-08

**Authors:** Xinyang Zhang

**Affiliations:** Dankook University, Yongin-si, Gyeonggi-do, Republic of Korea

**Keywords:** affective organizational commitment, employee performance, structural equation modeling, transformational leadership, China

## Abstract

**Objective:**

This study examined the association between transformational leadership and employee performance and tested whether affective organizational commitment serves as a mediating mechanism. The analysis focused on how meaning, recognition, and individualized support provided by leaders may strengthen employees’ emotional attachment to organizational goals and, in turn, relate to performance.

**Method:**

A quantitative cross sectional survey was conducted with 268 full time employees from manufacturing, service, and technology organizations in mainland China. Transformational leadership, affective organizational commitment, and employee performance were measured with adapted multi item scales. Structural equation modeling was used to assess the measurement and structural models, and the indirect association was evaluated using a bootstrap confidence interval. Anonymity, neutral wording, and confidential data handling were used to reduce response bias.

**Results:**

Transformational leadership was positively associated with employee performance and affective organizational commitment, while affective organizational commitment was positively associated with employee performance. The direct and indirect associations were both statistically significant. The results support partial mediation, indicating that commitment explains a substantial, but not exhaustive, part of the leadership performance relationship.

**Conclusion:**

Affective organizational commitment functions as a distinct psychological pathway through which transformational leadership is associated with employee performance. By isolating this attitudinal mechanism in Chinese organizations undergoing rapid organizational and technological change, the study clarifies why leader behaviors may support sustained performance while also showing that additional motivational and contextual pathways remain relevant.

## 1 Introduction

Employee performance is a central microfoundation of organizational competitiveness, particularly as firms respond to digital transformation, changing work arrangements, and intensified demands for adaptability. Transformational leadership can provide direction, meaning, and individualized support that encourage employees to extend effort beyond formal requirements (Buil et al., 2019). Performance nevertheless reflects an interaction among individual capabilities, work design, and organizational conditions, which means that leadership should be examined as one strategic influence within a broader system of employee functioning (Atatsi et al., 2019). The leadership performance association is therefore unlikely to operate through a single direct route and requires closer examination of the psychological states that connect leader behavior with employee responses (Qalati et al., 2022).

This study focuses on affective organizational commitment as a proximal psychological mechanism linking transformational leadership with employee performance. Affective commitment refers to employees’ emotional attachment to, identification with, and involvement in the organization. It is theoretically relevant because transformational leaders communicate collective purpose and recognize individual contributions, thereby strengthening the extent to which employees internalize organizational goals. Prior evidence links affective commitment with engagement and performance (Cesário and Chambel, 2017). Ribeiro et al. (2018) further showed that transformational leadership predicts affective commitment and performance. Although leadership studies often combine commitment with engagement, satisfaction, or empowerment, such multi mediator models make it difficult to determine the specific explanatory contribution of commitment (Aldossari and Alanizan, 2025). A focused model can therefore provide greater theoretical precision while recognizing that commitment is not the only possible pathway.

By concentrating on affective organizational commitment, the study separates an organization focused attitude from related mechanisms such as job satisfaction and engagement. The partial mediation result also defines the explanatory limit of commitment because the significant direct path suggests that leadership operates through additional motivational and work resource mechanisms. Evidence from Chinese manufacturing, service, and technology organizations further shows how leadership and commitment are associated during periods of organizational and technological change, when employees must adapt to new roles and capability requirements.

## 2 Literature review and theoretical background

### 2.1 Transformational leadership and employee performance

Transformational leadership is a form of leader behavior that motivates followers to pursue collective goals beyond immediate exchange relationships (Fahrudin, 2025). Burns (1978) distinguished transforming leadership from transactional exchange, while Bass (1985) developed the framework around idealized influence, inspirational motivation, intellectual stimulation, and individualized consideration. Through these behaviors, leaders communicate an attractive future, challenge routine assumptions, model responsible conduct, and attend to individual development (Hussain et al., 2024). Contemporary research indicates that these processes are associated with employee performance through identification, proactive behavior, and motivational resource mobilization (Buil et al., 2019).

Evidence across organizational settings generally supports a positive association between transformational leadership and employee performance, although the magnitude and mechanisms vary with job resources and organizational conditions (Hilton et al., 2023). Balwant et al. (2020) showed that engagement and job resources help explain why transformational leadership is related to service employee outcomes. Employee performance should therefore be treated as multidimensional, incorporating task accomplishment, adaptability, initiative, and cooperation rather than a single productivity indicator. The comprehensive performance framework developed by Na-Nan et al. (2018) is consistent with this approach. This multidimensional view is especially relevant in Chinese firms undergoing innovation and capability upgrading, where performance increasingly depends on learning, coordination, and adjustment to changing work requirements.

More recent evidence continues to support this relationship, with studies showing that transformational leadership is positively associated with employee performance across different organizational settings and that its effects may operate through attitudinal and motivational mechanisms (Ma and Chen, 2025).

Systematic review evidence also supports a positive association between transformational leadership and employee performance (Mahmud et al., 2023). Rony et al. (2023) report a similar pattern in a systematic literature review. Shang (2023) likewise synthesizes evidence linking transformational leadership with employee performance. Top et al. (2020) also report a positive relationship between transformational leadership and employee performance.

### 2.2 Affective organizational commitment as a mediator

Organizational commitment refers to a psychological bond connecting employees with their organization. The present study examines affective organizational commitment specifically, defined as emotional attachment, identification, and involvement, rather than continuance commitment based on the perceived cost of leaving. Employees with stronger affective commitment are more willing to invest discretionary effort, cooperate with colleagues, and persist in support of collective goals. Cesário and Chambel (2017) linked organizational commitment and engagement with employee performance. Transformational leadership is expected to strengthen affective commitment because shared meaning, recognition, trust, and individualized support make organizational membership more personally significant (Shidqi and Sudiro, 2025). Ribeiro et al. (2018) similarly reported positive associations among transformational leadership, affective commitment, and performance. More recent evidence has confirmed this pattern, as Jiatong et al. (2022) found that transformational leadership was positively related to both affective organizational commitment and job performance, reinforcing the relevance of affective commitment within contemporary leadership–performance models.

Mediation focused research is consistent with broader leadership models in which leader behavior shapes outcomes through employees’ motivational and relational states. Keskes et al. (2018) demonstrated that leader member exchange helps connect transformational leadership with organizational commitment. Park et al. (2021) further showed that affective commitment forms part of a wider sequence involving leadership, engagement, and performance. Studies that combine commitment with job satisfaction or engagement provide useful breadth, but they may obscure the unique explanatory role of commitment (Hendri, 2019). By treating affective commitment as the sole mediator, the present model tests whether emotional attachment to the organization constitutes a distinct pathway while allowing the remaining direct association to represent other unmeasured psychological and resource based mechanisms.

## 3 Conceptual framework and hypotheses

### 3.1 Conceptual framework

**Figure 1:**
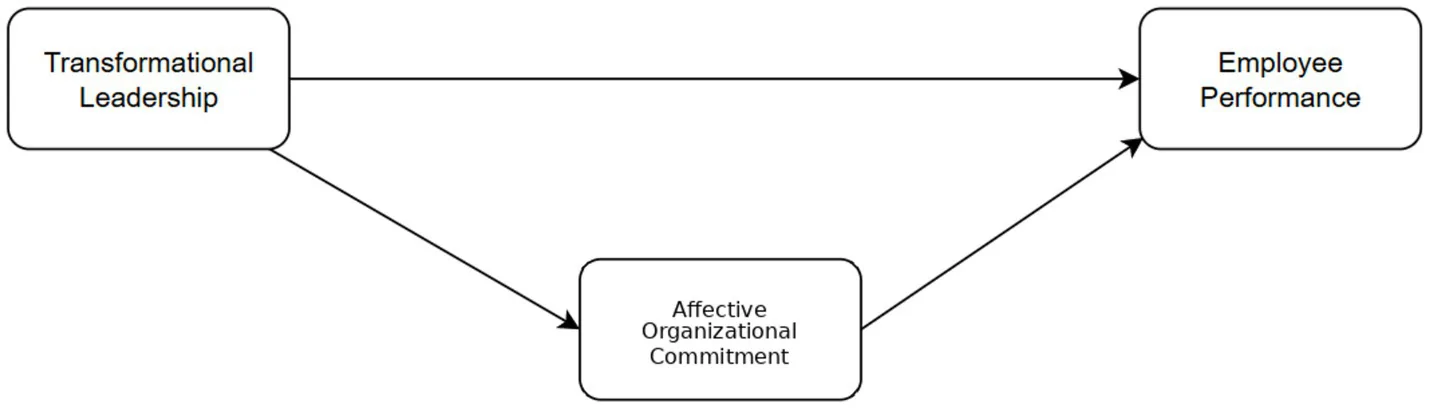
Conceptual model of the study.

The conceptual framework distinguishes a direct association from an indirect association through affective organizational commitment. The direct path reflects the possibility that transformational leaders influence coordination, goal clarity, confidence, and problem solving without first changing employees’ organizational attachment. The indirect path represents a psychological alignment process in which inspirational and individualized leader behaviors strengthen affective commitment, which then supports persistence, cooperation, and adaptive performance. Commitment is therefore treated as a proximal motivational state rather than a general evaluation of the job (Cesário and Chambel, 2017). This parsimonious specification improves theoretical precision because it estimates the unique mediating role of affective commitment while leaving open the possibility of other pathways, consistent with evidence that leadership can also operate through engagement and relational resources (Ribeiro et al., 2018). Recent empirical research further supports this framework by showing that affective commitment remains an important intervening mechanism between transformational leadership and employee performance, even when other motivational pathways are considered (Shidqi and Sudiro, 2025) (Figure 1).

### 3.2 Hypotheses development

Transformational leadership is expected to be positively associated with employee performance because it mobilizes intrinsic motivation and fosters a climate where employees feel empowered to exceed formal role requirements. Empirical evidence supports positive links between transformational leadership and performance through mechanisms such as identification and proactive orientation, indicating a robust foundation for a direct effect hypothesis (Buil et al., 2019). Recent studies have continued to report a positive relationship between transformational leadership and employee performance in contemporary organizational contexts (Farras et al., 2024). Systematic reviews further suggest that leadership is a key determinant among multiple factors shaping performance, which reinforces the plausibility of a positive association where leadership often structures work meaning and collective direction (Atatsi et al., 2019).

*H1*: Transformational leadership is positively related to employee performance (Bass, 1985).

Transformational leadership is expected to increase affective organizational commitment because inspirational motivation and individualized consideration signal leader support, value congruence, and future oriented opportunities, which foster emotional attachment to the organization. Evidence indicates that transformational leadership predicts affective commitment and that this attitudinal bond is strengthened when employees perceive leadership as supportive and meaningful (Ribeiro et al., 2018). Relational perspectives also imply that commitment can be strengthened through leadership that improves exchange quality and trust, which supports a positive association between transformational leadership and commitment (Keskes et al., 2018).

*H2*: Transformational leadership is positively related to affective organizational commitment (Ribeiro et al., 2018).

Affective organizational commitment is expected to be positively associated with employee performance because committed employees invest effort and persistence and are more likely to engage in cooperative and citizenship behaviors that enhance both individual and collective performance. Empirical research links commitment and engagement to performance outcomes and suggests that commitment contributes to sustained performance rather than temporary effort spikes (Cesário and Chambel, 2017). Evidence from mediated leadership models similarly implies that affective attachment supports job performance by motivating discretionary effort and strengthening alignment with performance standards (Eliyana et al., 2019).

*H3*: Affective organizational commitment is positively related to employee performance (Cesário and Chambel, 2017).

The mediation hypothesis proposes that affective organizational commitment transmits part of the association between transformational leadership and employee performance (Indriasari et al., 2023). Transformational leadership is theorized to build commitment by fostering identification and shared purpose, which then motivates performance beyond formal requirements (Bass, 1985). Empirical studies show patterns consistent with mediation, where leadership predicts commitment and performance and commitment related pathways explain part of the leadership performance association (Ribeiro et al., 2018). Recent evidence provides further support for this mediating mechanism, indicating that affective organizational commitment can transmit part of the effect of transformational leadership on employee performance (Shidqi and Sudiro, 2025).

*H4*: Affective organizational commitment mediates the relationship between transformational leadership and employee performance (Keskes et al., 2018).

## 4 Methodology

### 4.1 Research design and sample

This study examined whether affective organizational commitment mediates the association between transformational leadership and employee performance. A quantitative cross sectional survey design was adopted to test the measurement and structural models (Stockemer, 2019). The sampling frame comprised full time employees working in manufacturing, service, and technology organizations in mainland China. Participants were eligible when they had worked in their current organization for at least 6 months, which provided sufficient exposure to the behavior of their immediate supervisor and to organizational practices. A purposive, referral based sampling strategy was used because no complete cross industry employee sampling frame was available. Structural equation modeling was selected because the study involved latent constructs, measurement validation, and simultaneous estimation of direct and indirect associations (Blanco-Encomienda and Rosillo-Díaz, 2021).

The target sample was determined before data collection by considering model complexity, the number of latent variables and indicators, and the need for stable estimation of an indirect association. Samples above 200 are commonly considered adequate for moderately complex covariance based structural models when indicators have acceptable communalities and the model is identified (Cohen, 1988). A total of 350 questionnaires were distributed through an online survey platform. Of the 312 returned questionnaires, incomplete responses and cases failing attention checks were removed, leaving 268 valid cases. The achieved sample exceeded the planned minimum and provided an adequate observation to parameter ratio for the three construct confirmatory factor and structural models.

### 4.2 Measures and instrument development

The questionnaire used adapted rather than newly created measures. Transformational leadership was assessed with eight items representing idealized influence, inspirational motivation, intellectual stimulation, and individualized consideration, drawing on the Bass (1985) framework and wording used in later empirical leadership research. Affective organizational commitment was measured with six items adapted from the affective commitment operationalization used by Ribeiro et al. (2018). The construct was limited to emotional attachment, identification, and personal meaning; continuance commitment was not included. Employee performance was assessed with eight adapted items covering task completion, initiative, adaptability, cooperation, quality, and problem solving, based on the comprehensive performance scale developed by Na-Nan et al. (2018).

All items used a 5 point Likert response scale from strongly disagree to strongly agree. The questionnaire was translated into Chinese and reviewed during the pilot test for clarity and contextual appropriateness. Minor wording adjustments were made where necessary without changing the construct definitions or response scale. Reliability was assessed with Cronbach’s alpha and composite reliability (Gliem and Gliem, 2003). Convergent validity was evaluated using standardized loadings and average variance extracted, and discriminant validity was examined with the Fornell Larcker criterion and the heterotrait monotrait ratio. The measurement model was assessed before the structural paths and mediation relationship were interpreted (Table 1).

**Table 1:** Construct definitions and measurement sources.

Construct	Definition in this study	Example scale source in this article	Items	Scale
Transformational leadership	Leader behaviors that communicate collective purpose, stimulate new thinking, model responsible conduct, and support individual development	Items adapted from Bass (1985) and later transformational leadership research	8	5 point Likert
Affective organizational commitment	Employees’ affective attachment, identification, and emotional involvement with the organization and its goals	Affective commitment items adapted from Ribeiro et al. (2018)	6	5 point Likert
Employee performance	Employee effectiveness in task execution, initiative, cooperation, adaptability, work quality, and problem solving	Items adapted from Na-Nan et al. (2018)	8	5 point Likert

The transformational leadership measure followed the four dimensional conceptualization of Bass (1985) and was adapted for employees’ ratings of their immediate supervisors. The affective organizational commitment items were adapted from Ribeiro et al. (2018) and consistently represented emotional attachment and identification. Employee performance items were adapted from the validated comprehensive performance scale of Na-Nan et al. (2018) (Table 2).

**Table 2:** Survey items used in the study.

Code	Item statement
Transformational leadership (TL)	
TL1	My supervisor communicates a clear and inspiring vision for the future.
TL2	My supervisor encourages me to view problems from new perspectives.
TL3	My supervisor treats employees as individuals with different needs and abilities.
TL4	My supervisor sets an example of ethical and responsible behavior.
TL5	My supervisor expresses confidence that goals will be achieved.
TL6	My supervisor challenges me to think critically about routine work practices.
TL7	My supervisor supports my development through coaching and feedback.
TL8	My supervisor motivates me to go beyond what is expected in my job.
Affective organizational commitment (OC)	
OC1	I feel a strong sense of belonging to my organization.
OC2	I feel emotionally attached to my organization.
OC3	I am proud to tell others that I am part of this organization.
OC4	I share the values and goals of my organization.
OC5	I am willing to put in extra effort to help this organization succeed.
OC6	This organization has a great deal of personal meaning for me.
Employee performance (EP)	
EP1	I consistently meet the performance standards of my job.
EP2	I complete my tasks accurately and on time.
EP3	I take initiative to solve work related problems.
EP4	I adapt quickly when work priorities change.
EP5	I cooperate effectively with colleagues to achieve shared goals.
EP6	I maintain high quality in my work even under pressure.
EP7	I contribute ideas that improve work processes or outcomes.
EP8	I handle unexpected difficulties in my work effectively.

The item wording was adapted to the Chinese organizational context while retaining the conceptual content of the source measures. Transformational leadership items represented the four dimensions specified by Bass (1985). All commitment items assessed affective attachment, identification, and personal meaning in accordance with Ribeiro et al. (2018). Performance items represented task, adaptive, and contextual performance domains identified by Na-Nan et al. (2018).

### 4.3 Data collection procedures and statistical analysis plan

Data were collected from full time employees in manufacturing, service, and technology organizations in mainland China through an anonymous web based questionnaire distributed through professional networks and referrals. The survey information page explained the study purpose, the confidential treatment and academic use of responses, the voluntary nature of participation, and the right to discontinue without penalty. Participants indicated informed consent before accessing the questionnaire. No names, contact details, or other personally identifying information were collected, and individual responses were not shared with employers. Neutral wording and anonymity were used to reduce evaluation apprehension and socially desirable responding.

A pilot test with 30 employees assessed item clarity, relevance, and completion time. Minor wording changes were made where literal translation produced ambiguity, but no construct definition or response scale was changed. The leadership items referred consistently to the respondent’s immediate supervisor. The commitment items were restricted to affective organizational commitment and did not assess continuance or normative commitment. The performance items covered task execution, initiative, adaptability, cooperation, and work quality.

A two stage structural equation modeling procedure was applied. The measurement model was evaluated before the structural relationships were interpreted, following established SEM practice (Blanco-Encomienda and Rosillo-Díaz, 2021). Reliability, convergent validity, and discriminant validity were assessed using the criteria specified above. All 22 planned indicators were retained because their standardized loadings were acceptable and their content remained aligned with the construct definitions. The structural model was then used to test the direct paths and the indirect association through affective organizational commitment.

Data analysis was conducted using IBM SPSS Amos 26.0. The measurement and structural models were estimated using the maximum likelihood estimation method. The measurement model was first assessed through confirmatory factor analysis, followed by the estimation of the hypothesized structural paths. The mediating effect of organizational commitment was examined using a nonparametric bootstrapping procedure with 5,000 resamples. Bias-corrected 95% confidence intervals were calculated for the indirect effect, and mediation was considered statistically significant when the confidence interval did not include zero.

Mediation was evaluated from the direct, indirect, and total standardized associations. The indirect association was judged significant when its bootstrap confidence interval excluded zero. Partial mediation was inferred because the indirect association was significant and the direct association remained significant after affective organizational commitment was included. Anonymity, neutral wording, confidential data handling, and separate questionnaire sections were used to reduce common method concerns. Because the three variables were collected from the same respondents at one time point, common method bias could not be eliminated and was considered when interpreting the findings.

## 5 Results

### 5.1 Sample profile

A total of 312 questionnaires were returned. After incomplete responses and cases failing attention checks were removed, 268 valid observations remained for analysis. The final sample exceeded the planned minimum for the three construct structural equation model and provided sufficient observations for stable estimation. Participants represented manufacturing, service, and technology organizations, as well as nonmanagerial and managerial positions. This heterogeneity supports examination of leadership perceptions across different job and industry contexts while maintaining a specific focus on Chinese organizations (Table 3).

**Table 3:** Demographic characteristics of respondents (*N* = 268).

Characteristic	Category	Frequency	Percentage
Gender	Male	152	56.7
	Female	116	43.3
Age	18–24	41	15.3
	25–34	123	45.9
	35–44	73	27.2
	45+	31	11.6
Education	High school or below	28	10.4
	Bachelor	156	58.2
	Master or above	84	31.3
Tenure	0.5–2 years	62	23.1
	3–5 years	88	32.8
	6–10 years	74	27.6
	11 + years	44	16.4
Industry	Manufacturing	92	34.3
	Services	103	38.4
	Technology	73	27.2
Position	Non managerial	161	60.1
	Managerial	107	39.9

The sample was concentrated among early and middle career employees but included meaningful variation in age, education, tenure, industry, and position. This profile is relevant to Chinese firms undergoing capability upgrading because employees in such settings are frequently required to learn, coordinate, and adapt under organizational change (Xu and Rosli, 2025). The broad tenure distribution also provides variation in the opportunity to develop affective attachment. The sample should nevertheless be interpreted as a purposive cross industry employee sample rather than as statistically representative of the entire Chinese workforce.

### 5.2 Descriptive statistics and reliability

The three constructs had moderate to high mean scores, indicating that respondents generally reported favorable leadership, commitment, and performance perceptions. The standard deviations showed sufficient variability for correlational and structural analysis. Because all measures were self reported, these descriptive levels should not be interpreted as objective organizational performance or as evidence that favorable perceptions were uniformly distributed across firms (Table 4).

**Table 4:** Descriptive statistics and internal consistency (*N* = 268).

Construct	Items	Mean	SD	Cronbach’s alpha
Transformational leadership (TL)	8	3.74	0.69	0.91
Affective organizational commitment (OC)	6	3.61	0.66	0.89
Employee performance (EP)	8	3.79	0.62	0.92

Cronbach’s alpha values ranged from 0.89 to 0.92, indicating strong internal consistency. These values support the use of the item sets as latent construct indicators, but high reliability alone does not establish construct validity or eliminate common method bias. The reliability evidence was therefore considered together with factor loadings, composite reliability, average variance extracted, discriminant validity, and alternative model comparisons before the structural associations were interpreted.

### 5.3 Measurement model assessment

Confirmatory factor analysis was conducted for the hypothesized three factor model comprising transformational leadership, affective organizational commitment, and employee performance. The model provided acceptable overall fit. Standardized loadings, composite reliability, average variance extracted, the Fornell Larcker criterion, and the heterotrait monotrait ratio were considered together when evaluating construct quality. The results support the empirical distinction among the three constructs, although the single source design still permits common response tendencies (Table 5).

**Table 5:** Standardized factor loadings from CFA (*N* = 268).

Construct	Item	Loading
TL	TL1	0.82
	TL2	0.84
	TL3	0.79
	TL4	0.77
	TL5	0.81
	TL6	0.83
	TL7	0.80
	TL8	0.86
OC	OC1	0.81
	OC2	0.84
	OC3	0.79
	OC4	0.82
	OC5	0.77
	OC6	0.75
EP	EP1	0.78
	EP2	0.82
	EP3	0.80
	EP4	0.76
	EP5	0.79
	EP6	0.83
	EP7	0.77
	EP8	0.81

Standardized loadings ranged from 0.75 to 0.86 and were adequate for all retained items. All 22 planned indicators remained in the measurement model, and each item continued to represent the construct domain specified during instrument development. The loading pattern therefore supports convergent validity without changing the number or conceptual coverage of the items (Table 6).

**Table 6:** Composite reliability and convergent validity (*N* = 268).

Construct	CR	AVE
Transformational leadership (TL)	0.93	0.62
Affective organizational commitment (OC)	0.90	0.60
Employee performance (EP)	0.93	0.63

Composite reliability values ranged from 0.90 to 0.93 and average variance extracted values ranged from 0.60 to 0.63. Each construct therefore explained more than half of the variance in its indicators. These results support convergent validity, but they were interpreted alongside discriminant validity and model comparison evidence rather than as independent proof that the measurement model was free from misspecification.

Discriminant validity was evaluated with the Fornell Larcker criterion and the heterotrait monotrait ratio. This assessment was particularly important because employees who evaluate their supervisors positively may also report stronger commitment and better performance. Demonstrating empirical distinction reduces the likelihood that the mediation result merely reflects overlapping item content or a generalized favorable response tendency (Table 7).

**Table 7:** Correlations and Fornell Larcker discriminant validity (*N* = 268).

Construct	TL	OC	EP
TL	0.79		
OC	0.63	0.77	
EP	0.58	0.61	0.79

Diagonal elements are the square root of AVE. Off diagonal elements are correlations.

For each construct, the square root of average variance extracted exceeded its correlations with the other constructs. The correlations were substantial but below levels suggesting redundancy. These results support treating transformational leadership, affective organizational commitment, and employee performance as related but distinct latent variables (Table 8).

**Table 8:** HTMT ratios (*N* = 268).

Pair	HTMT
TL–OC	0.74
TL–EP	0.69
OC–EP	0.72

All heterotrait monotrait ratios were below conservative thresholds. Together with the Fornell Larcker results and the acceptable factor loadings, this evidence supports discriminant validity. Affective organizational commitment can therefore be interpreted as a distinct mediating construct rather than as another label for favorable leadership perceptions. The self report design nevertheless requires caution because discriminant validity does not eliminate common method variance.

### 5.4 Structural model and hypothesis testing

The structural model specified transformational leadership as directly associated with employee performance and indirectly associated with it through affective organizational commitment. The model retained the validated three factor measurement structure and accounted for measurement error while estimating the paths simultaneously. The coefficients are interpreted as cross sectional associations rather than causal effects (Table 9).

**Table 9:** Structural model fit indices (*N* = 268).

Fit index	Value
χ^2^/df	2.21
CFI	0.95
TLI	0.94
RMSEA	0.067
SRMR	0.048

The chi square ratio, comparative fit index, Tucker Lewis index, root mean square error of approximation, and standardized root mean square residual jointly indicated acceptable structural model fit. The conclusion was based on the pattern across several indices rather than on a single cutoff, supporting interpretation of the hypothesized paths within the specified three construct model (Table 10).

**Table 10:** Hypothesis testing results (standardized estimates, *N* = 268).

Path	*β*	SE	*t*	*p*
H1: TL → EP	0.26	0.07	3.71	< 0.001
H2: TL → OC	0.63	0.06	10.50	< 0.001
H3: OC → EP	0.45	0.08	5.63	< 0.001

Transformational leadership was positively associated with employee performance, supporting H1. It was also strongly associated with affective organizational commitment, supporting H2. Affective organizational commitment was positively associated with employee performance, supporting H3. These results are consistent with the view that transformational leaders provide meaning, recognition, and developmental support that strengthen emotional attachment to the organization, while committed employees invest greater effort and cooperation. The retained direct association also indicates that commitment does not exhaust the psychological and contextual processes linking leadership with performance.

### 5.5 Mediation analysis

The indirect association was evaluated using a bootstrap confidence interval. The confidence interval for the path from transformational leadership to employee performance through affective organizational commitment did not include zero. This procedure avoids reliance on a normal sampling distribution for the product of coefficients and provides the basis for evaluating H4 (Table 11).

**Table 11:** Direct, indirect, and total associations (bootstrap results, *N* = 268).

Effect	Estimate	95% CI lower	95% CI upper	Result
Direct association TL → EP	0.26	0.12	0.39	Significant
Indirect association TL → OC → EP	0.28	0.18	0.40	Significant
Total association TL → EP	0.54	0.41	0.66	Significant

The direct, indirect, and total associations were all statistically significant. Because the direct path remained significant after affective organizational commitment was included, the result supports partial mediation. Commitment therefore transmits a substantial part of the leadership performance association but does not constitute the only mechanism. The retained direct association is consistent with additional pathways involving engagement, job resources, psychological safety, and proactive behavior (Motyka, 2018).

### 5.6 Common method and covariate considerations

Common method bias remains a potential limitation because leadership, commitment, and performance were reported by the same respondents at one point in time. Procedural safeguards included anonymity, neutral wording, confidential treatment of responses, and separate construct sections. The acceptable three factor measurement model and discriminant validity results reduce concern about simple construct overlap, but they do not rule out a shared response tendency. Tenure and position were described in the sample profile but were not included as covariates in the reported structural model. The path coefficients should therefore be interpreted as unadjusted associations, and demographic differences cannot be considered eliminated.

## 6 Discussion

The findings support a model in which transformational leadership is associated with employee performance through both direct and commitment based pathways. The direct association is consistent with the capacity of transformational leaders to clarify goals, stimulate problem solving, and strengthen confidence in task accomplishment. The indirect association provides a more specific explanation: inspirational and individualized leader behaviors are related to stronger affective attachment, and this attachment is associated with persistence, cooperation, and adaptive performance. The partial mediation pattern is theoretically important because it demonstrates that affective commitment is a substantial mechanism without treating it as a complete explanation of the leadership performance relationship.

This pattern is consistent with more recent evidence. Jiatong et al. (2022) similarly reported positive relationships among transformational leadership, affective organizational commitment, and job performance, although their model emphasized employee engagement as an additional mediating mechanism. More recently, Shidqi and Sudiro (2025) also identified affective commitment as an important pathway connecting transformational leadership with employee performance. The present findings therefore reinforce recent evidence on the importance of affective commitment while showing that this mechanism remains significant when examined as the focal mediator in a more parsimonious model.

The strong association between transformational leadership and affective organizational commitment suggests that employees respond not only to formal managerial direction but also to the meaning and relational quality conveyed by leaders. Recognition, individualized support, and collective purpose can make organizational membership personally significant, thereby increasing emotional attachment. This interpretation extends beyond a restatement of the coefficients because it identifies affective commitment as the attitudinal state through which leadership signals are translated into sustained willingness to contribute. It also differentiates commitment from engagement and job satisfaction, which concern energetic involvement and job evaluation rather than attachment to the organization itself.

The positive association between affective organizational commitment and performance indicates that emotional attachment has behavioral relevance. Employees who identify with organizational goals are more likely to persist under difficulty, coordinate with colleagues, and maintain work quality during change. This mechanism is particularly relevant to workforce sustainability because commitment may support continuity, knowledge retention, and cooperative adaptation rather than only short term output. The findings also connect the model with employee wellbeing and psychological safety: leadership that communicates support and respect may strengthen commitment without relying exclusively on pressure or transactional control.

The Chinese context helps refine, rather than merely replicate, the proposed mechanism. Many sampled organizations were operating amid technological upgrading and organizational change, conditions in which employees face uncertainty about roles, skills, and future expectations. Transformational leadership may be especially salient in this setting because clear collective purpose and individualized recognition can reduce uncertainty and align personal effort with organizational transformation. At the same time, the cross sectional design does not establish that national culture caused the observed relationships. Comparative research across institutional and cultural settings is needed to determine whether hierarchical relations, collective goal orientation, or employment stability strengthen or weaken the commitment pathway.

### 6.1 Theoretical implications

The findings refine the role of affective organizational commitment in leadership research. Studies often combine commitment with engagement, satisfaction, empowerment, or leader member exchange, making it difficult to identify the contribution of an organization focused attitude. In the present model, emotional attachment explains a substantial portion of the association between transformational leadership and performance while remaining empirically distinct from both constructs.

The significant direct path places an important limit on the mediating explanation. Affective organizational commitment is one pathway, but transformational leadership may also support performance through engagement, psychological safety, proactive orientation, learning, and access to job resources (Norawati et al., 2025). The results therefore identify both what commitment explains and what remains outside the scope of the model.

The Chinese evidence adds contextual detail to the model. Meaning, recognition, and individualized support were associated with commitment and performance in manufacturing, service, and technology organizations facing capability and digital transformation. The results do not establish cultural universality. They indicate instead that institutional arrangements, hierarchical expectations, collective goal orientation, and employment conditions may influence the strength of the commitment pathway and warrant direct comparative examination.

### 6.2 Practical implications

Organizations should develop transformational leadership capabilities through training in goal communication, intellectual stimulation, individualized coaching, and constructive recognition (Siahaan et al., 2025). These practices are particularly important during digital transformation and hybrid work because employees need clarity, autonomy, and psychological safety when routines and skill requirements change. Leadership development should therefore be evaluated not only by immediate output but also by whether it strengthens trust, learning, and sustainable performance.

Organizations should also treat affective commitment as an outcome of credible employment practices rather than as a demand placed on employees. Fair treatment, transparent communication, development opportunities, and meaningful participation can reinforce emotional attachment and reduce withdrawal intentions (Abid et al., 2019). Monitoring commitment alongside wellbeing and workload is important because sustainable commitment should be based on identification and support, not on pressure to remain or perceived costs of leaving.

Managers can further strengthen workforce sustainability by creating work contexts that support cooperation, recognition, and adaptive learning. In hybrid or technology enabled workplaces, regular developmental feedback and inclusive communication can maintain employees’ connection with organizational goals despite reduced face to face contact. Such practices may help retain knowledge, support psychological safety, and improve coordination during organizational change. The findings therefore have relevance beyond conventional leadership training and extend to human resource systems designed for employee wellbeing and long term organizational resilience.

### 6.3 Limitations and future research

The cross sectional design limits causal inference. Although the theoretical ordering places transformational leadership before affective commitment and performance, the data cannot exclude reverse or reciprocal relationships. Employees who perform well or feel strongly attached may evaluate their supervisors more favorably. Longitudinal, experimental, and time separated designs are needed to examine temporal ordering and changes in commitment.

All focal variables, including employee performance, were reported by the same respondents at one point in time. Anonymity, neutral wording, confidential data handling, and separate construct sections reduced evaluation apprehension and obvious item overlap, but they cannot eliminate common method bias or self enhancement in performance ratings. The reported model also did not adjust the focal paths for tenure or position. Future studies should obtain supervisor ratings, objective indicators, or matched multisource data, separate predictor, mediator, and outcome measurement over time, and examine whether demographic and organizational covariates alter the estimated associations.

The purposive referral sample also limits population generalizability, and the broad industry categories do not capture firm ownership, region, organizational size, hybrid work intensity, or digital maturity. Future research should compare state owned and private firms, different Chinese regions, and organizations at different stages of digital transformation. Cross cultural studies can test whether hierarchy, collectivism, employment security, and institutional support condition the leadership commitment pathway. Evidence that employee engagement mediates relationships between work attitudes and performance further supports examining engagement alongside commitment (Riyanto et al., 2021). Additional mediators such as engagement, psychological safety, thriving, and job resources should be examined in parallel models to determine their unique and combined explanatory value (Arifin et al., 2019).

## 7 Conclusion

This study examined the direct and indirect associations among transformational leadership, affective organizational commitment, and employee performance in a sample of 268 employees in mainland China. Transformational leadership was positively associated with both commitment and performance, and affective organizational commitment was positively associated with performance. The significant direct and indirect associations support partial mediation. Affective commitment therefore represents an important, but not exclusive, psychological mechanism linking leadership with employee performance.

The study clarifies the role and limits of affective organizational commitment within a focused mediation model and interprets this relationship in Chinese organizations undergoing technological and organizational change. The findings suggest that leadership development should be combined with fair, supportive, and development oriented employment practices that strengthen emotional attachment, psychological safety, and sustainable performance. The cross sectional and self reported evidence does not support strong causal or universal conclusions, which should be examined through longitudinal, multisource, and cross cultural research.

## Data Availability

The original contributions presented in the study are included in the article/supplementary material, further inquiries can be directed to the corresponding author.
